# Exertional Hyponatremia Among U.S. Active Component Service Members, 2021–2025

**Published:** 2026-05-20

## Abstract

Exertional hyponatremia, also known as exercise-associated hyponatremia, is a fluid electrolyte disorder defined by a low sodium level in the blood (below 135 mEq/L). Exertional hyponatremia results from excessive fluid intake that dilutes serum sodium, impairing neurological and other organ functions. Hyponatremia can be fatal if not detected early and managed properly. From 2021 through 2025, 609 cases of exertional hyponatremia were diagnosed among U.S. active component service members (ACSMs), with an overall incidence rate of 9.2 cases per 100,000 person-years (p-yrs). In 2025, 106 cases of exertional hyponatremia were diagnosed among ACSMs, resulting in an incidence rate of 8.1 per 100,000 p-yrs. The highest incidence rates in 2025 were observed among males, individuals ages 35-39 years, non-Hispanic White service members, those in health care occupations, and personnel stationed in the western U.S. Notably, between 2024 and 2025 the Marine Corps and recruit populations showed a sharp decline in cases. From 2021 to 2023, annual rates of incident exertional hyponatremia diagnoses increased, peaking in 2023 (11.6 per 100,000 p-yrs) and then decreased to 8.1 cases per 100,000 p-yrs in 2025. Although the incidence of exertional hyponatremia is decreasing, continued monitoring and specialized prevention strategies are critical, as the associated risk factors may affect individuals differently.

**What are the new findings?**

Incidence rates of exertional hyponatremia decreased from 10.6 per 100,000 people per year in 2024 to 8.1 per 100,000 in 2025. Rates increased sharply in the 35-39-years age group, however, while decreasing sharply among those younger than age 20 years, ages 25-29 years, and in the Marine Corps.

**What is the impact on readiness and force health protection?**

The recent decline in cases of exertional hyponatremia is positive, but shifting rates among demographic groups show that risks are still dynamic. Due to the fact exertional hyponatremia can be fatal, commanders and trainers should prioritize enforcement of proper hydration protocols, maintain vigilance to identify early symptoms, and if necessary ensure immediate, prescribed intervention.

Exertional hyponatremia is a fluid electrolyte disorder resulting from excessive consumption of hypotonic fluids such as water. Although exertional hyponatremia is relatively rare, it can be fatal if not detected early and managed properly. Exertional hyponatremia is caused by increased consumption of hypotonic fluids, such as water or sports drinks, before or during strenuous physical activity, such as prolonged military field training and combat operations. Active component military personnel are particularly susceptible to fluid and electrolyte imbalances due to intense exertion and demanding physical activities. ^1 , 2^ Key individual risk factors besides excessive fluid intake include exercise lasting more than 4 hours, inadequate training, and a high or low body mass index. ^3^ Risk of exertional hyponatremia is influenced by a range of factors, including the duration and type of activity—from military exercises to endurance races—as well as environmental conditions such as heat stress and water availability.

The severity and onset of exertional hyponatremia symptoms depend on the rate and degree of the decrease in serum sodium from normal levels. When a serum or plasma sodium concentration is less than 135 milliequivalents per liter (mEq/L) within 24 hours after prolonged physical activity, hyponatremia or exertional hyponatremia occur. Normal plasma sodium concentration (Na+) is closely regulated between 135 and 145 mEq/L to maintain proper cell size and function. ^1^ Excessive intake of sodium will stimulate thirst to increase body water to maintain normal sodium serum concentration. ^4 , 5^ Exertional hyponatremia can also be caused by inappropriate secretion of a non-osmotic antidiuretic hormone due to physical exertion, resulting in increased total body and free water retention. ^6^

Symptoms of exertional hyponatremia, which can manifest during or after physical activity, range from mild to life-threatening. Mild symptoms include lightheadedness, malaise, fatigue, irritability, weakness, headache, nausea, and reduced urine excretion. Severe symptoms can escalate to vomiting, oliguria or anuria, altered mental status, collapse, seizures, coma, and death. ^6^ Hyponatremia is treated primarily by managing the underlying cause and free water restriction, ^7^ focusing on pre-hospital care through rapid on-site emergency medical service assessment, as well as emergency and inpatient hospital management. ^8^ Depending on the physical demands of military operations and prevailing environmental conditions, replacement fluid composition may vary. ^9^

Hyponatremia is particularly problematic in the military, where it can be mistaken for an exertional heat illness (EHI), such as heat exhaustion or heat stroke, with corresponding symptomology that makes differential diagnosis difficult. ^8^ Exertional hyponatremia must be differentiated from EHI to avoid inappropriate treatment and adverse outcomes. ^10^ Failure to differentiate between these conditions can lead to incorrect treatment including overhydration, which risks severe and potentially permanent neurological damage. ^11^

The fundamental characteristics of military operations, such as long-term military training and combat operations in extreme environmental conditions, mean that exertional hyponatremia continues to pose a health risk to U.S. military personnel, with the potential for significantly reducing performance and combat effectiveness. There is growing evidence that hyponatremia is associated in various clinical settings and diseases with increased morbidity, mortality, and health costs. ^12 - 14^ *MSMR* annually summarizes the numbers, rates, trends, risk factors, and locations of exertional heat injury occurrences including exertional hyponatremia. This report includes updated surveillance data from 2021 through 2025. Additional information about the definition, causes, and prevention of exertional hyponatremia can be found in previous issues of *MSMR* . ^14^ This report summarizes the frequency, rates, trends, demographic, geographic location, and military characteristics of exertional hyponatremia cases among U.S. active component service members (ACSMs) from 2021 to 2025.

## Methods

The surveillance period ranged from January 2021 through December 2025 and included all individuals who served in the active component of the U.S. Army, Navy, Air Force, Marine Corps, Space Force, or Coast Guard. All data used to determine incident exertional hyponatremia diagnoses were derived from records routinely collected and maintained in the Defense Medical Surveillance System (DMSS). Those records document both ambulatory encounters and hospitalizations of U.S. Armed Forces ACSMs in fixed military and civilian (if reimbursed through the Military Health System) hospitals and clinics worldwide.

A case of exertional hyponatremia was defined as an individual with 1) a hospitalization or ambulatory visit with a primary (first-listed) diagnosis of “hypo-osmolality and/or hyponatremia” (International Classification of Diseases, 9th and 10th revisions, ICD-9: 276.1, ICD-10: E87.1) and no other illness or injury-specific diagnoses (ICD-9: 001–999, ICD-10: ‘A’–‘U’) in any diagnostic position or 2) both a diagnosis of ‘hypoosmolality and/or hyponatremia’ (ICD-9: 276.1, ICD-10: E87.1) and at least 1 of the following within the first 3 diagnostic positions (dx1–dx3): ‘fluid overload’ (ICD-9: 276.9; ICD-10: E87.70, E87.79), ‘alteration of consciousness’ (ICD-9: 780.0*, ICD-10: R40.*), ‘convulsions’ (ICD-9: 780.39, ICD-10: R56.9), ‘altered mental status’ (ICD-9: 780.97, ICD-10: R41.82), ‘effects of heat/light’ (ICD-9: 992.0–992.9, ICD-10: T67.0*– T67.9*), or ‘rhabdomyolysis’ (ICD-9: 728.88, ICD-10: M62.82). ^15^

Medical encounters were excluded from case-defining events if the associated records listed diagnoses in any diagnostic position that included alcohol or illicit drug abuse; psychosis, depression, or other major mental disorders; endocrine disorders; kidney diseases; intestinal infectious diseases; cancers; major traumatic injuries; or complications of medical care. An individual could be considered a case of exertional hyponatremia only once per calendar year. Incidence rates were calculated as cases of hyponatremia per 100,000 person-years (p-yrs) of active component service.

For health surveillance purposes, recruits were identified as active component members assigned to service-specific training locations during coincident service-specific basic training periods. Recruits were considered as a separate category of enlisted service members in summaries of exertional hyponatremia by military grade overall. Incidence rates reported in this update represent unadjusted rates.

## Results

**FIGURE 1.**
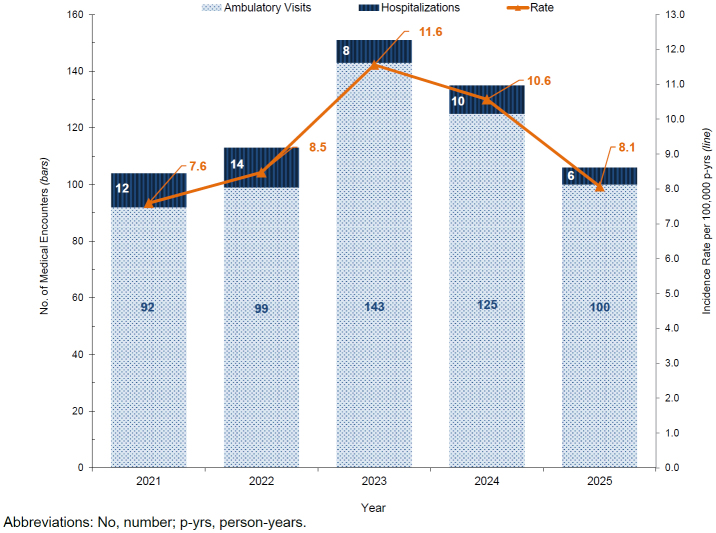
Annual Incident Cases and Rates of Exertional Hyponatremia, Active Component, U.S. Armed Forces, 2021–2025

In 2025, a total of 106 cases of exertional hyponatremia were identified among ACSMs, corresponding to an incidence rate (IR) of 8.1 per 100,000 p-yrs, a noteworthy decrease from 10.6 per 100,000 p-yrs in 2024. The IR of 8.1 recorded in 2025 represents the lowest annual rate for the entire 2021–2025 surveillance period, just 2 years after the peak in incidence, 11.6 per 100,000 p-yrs, in 2023. From 2021 through 2025, 609 incident cases of exertional hyponatremia were reported among ACSMs, resulting in an overall IR of 9.2 cases per 100,000 p-yrs. During the 5-year surveillance period, 91.8% (n=559) of all cases were diagnosed and treated without hospitalization (data not shown). **Figure 1** displays the annual incident cases and rates among ACSMs.

Although the Marine Corps had the highest overall IR (12.6 per 100,000 p-yrs) from 2021 to 2025, the Marine Corps rate dropped significantly between 2024 and 2025, from 17.0 to 7.6 per 100,000 p-yrs. While the annual IRs in the Army had been trending upward from 2022 to 2024, the IR for this service branch also declined in 2025 **(Figure 3)** .

**FIGURE 2.**
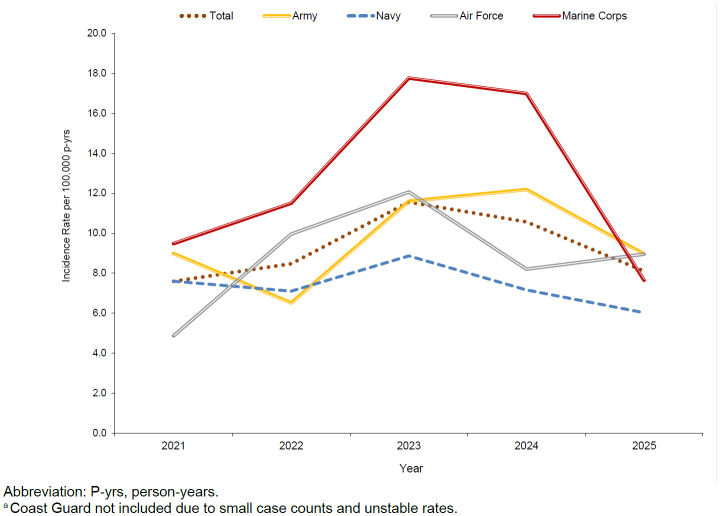
Annual Incidence Rates of Exertional Hyponatremia by Branch of Service ^a^ , Active Component, U.S. Armed Forces, 2021–2025

**FIGURE 3.**
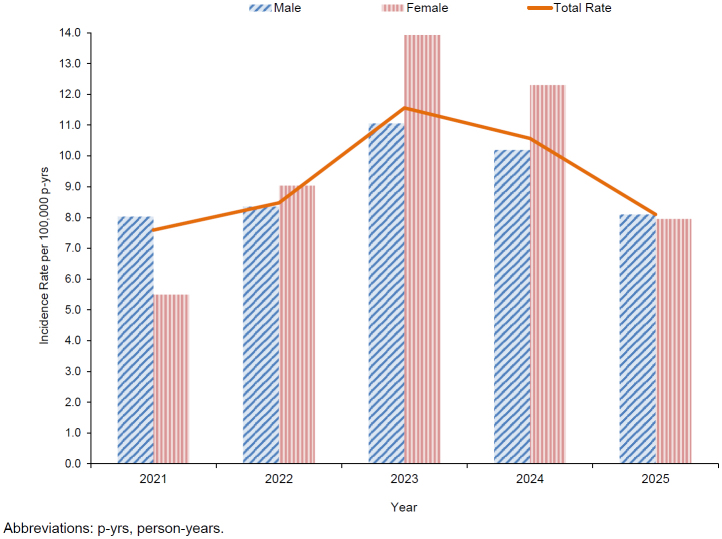
Annual Incident Rates of Exertional Hyponatremia by Sex, Active Component, U.S. Armed Forces, 2021–2025

Incidence of exertional hyponatremia among recruits showed a sharp decline, of 64.8%, from 2024 to 2025, dropping from 49.9 cases per 100,000 p-yrs to 17.6 per 100,000 p-yrs. Only 5 cases of hyponatremia were recorded among recruits in 2025 **(Table 1)** . The trend aligns with the overall pattern of case occurrence, which peaked in 2023 before shifting downward (data not shown).

**TABLE 1.** Incident Cases ^a^ and Ratesb of Exertional Hyponatremia, Active Component, U.S. Armed Forces, 2021–2025

	2025		Total, 2021–2025	
	No. ^a^	Rate ^b^	No. ^a^	Rate ^b^
Total	106	8.1	609	9.2
Sex				
Male	87	8.1	496	9.1
Female	19	8.0	113	9.7
Age, *y*				
<20	7	7.8	55	12.9
20–24	15	3.8	131	6.5
25–29	14	4.5	110	7.1
30–34	15	7.0	83	7.7
35–39	31	18.3	92	11.0
40+	24	17.3	138	20.3
Race and ethnicity				
White, non-Hispanic	58	8.8	344	9.9
Black, non-Hispanic	17	7.7	107	10.1
Hispanic	16	5.8	93	7.3
Other, unknown	15	9.2	65	8.0
Branch of service				
Army	40	9.0	218	9.6
Navy	20	6.0	122	7.3
Air Force	29	9.0	142	8.8
Marine Corps	13	7.6	108	12.6
Coast Guard	4	9.7	19	9.5
Military rank				
Enlisted	73	7.0	390	7.4
Officer	28	11.5	164	13.5
Recruit	5	17.6	55	43.5
Military occupation				
Combat-specific ^c^	13	8.1	90	10.6
Motor transport	0.0	9	4.1
Pilot, air crew	5	11.5	20	8.8
Repair, engineering	18	5.1	116	6.2
Communications, intelligence	22	8.2	129	9.3
Health care	15	14.4	55	10.3
Other	33	9.7	190	12.7
Home of record				
Midwest	14	7.2	100	9.7
Northeast	12	7.7	77	9.7
South	42	7.2	259	9.0
West	31	10.2	130	8.5
Other, unknown	7	9.0	43	11.5

Abbreviation: No., number; *y* , years

a Count 1 per person per year

b Cases per 100,000 person-years

c Includes infantry, artillery, combat engineering, armor.

Source: Defense Medical Surveillance System (DMSS) as of 16MAR2026

Prepared by Armed Forces Health Surveillance Division (AFHSD)

In 2025, the highest occupational IRs were found in the health care (14.4 per 100,000 p-yrs) and pilot and air crew (11.5 per 100,000 p-yrs) military professions; no cases were reported in motor transport. Although the overall trend has declined since its peak in 2023, the rates for health care as well as pilot and air crew occupations have continued to rise (data not shown).

In 2025, service members ages 35-39 years had the highest IR for exertional hyponatremia, followed by those ages 40 years and older (18.3 and 17.3 per 100,000 p-yrs, respectively). The IR for the 35-39-years age group increased noticeably in 2025 compared to 2024, to 18.3 from 7.2 per 100,000 p-yrs, respectively. Since 2023, the incidence of exertional hyponatremia has decreased for all age groups except the 30-39-years group (data not shown).

For both male and female ACSMs, cases of exertional hyponatremia declined in 2025 from the previous year. There was no significant difference in the annual IRs of male and female ACSMs in 2025: 8.1 and 8.0 per 100,000 p-yrs, respectively. While the IRs of hyponatremia for both sexes decreased from 2024 to 2025, the drop was more significant for women. The female IR fell by 35.0% (from 12.3 to 8.0 per 100,000 p-yrs), compared to a 20.6% decrease in the male IR (from 10.2 to 8.1 per 100,000 p-yrs). This higher variability in female rates was evident in all observed periods **(Figure 3)** .

From 2024 to 2025, the IRs of exertional hyponatremia declined among all racial and ethnic groups, excluding the ‘other or unknown’ category. The most substantial decrease was among Hispanic ACSMs, followed by non-Hispanic White and non-Hispanic Black service members. Notably, the trend for non-Hispanic White ACSMs had been increasing until 2024, when it began to decline. For all other ethnic groups, the decline in IRs started in 2023 (data not shown).

ACSMs stationed in the western U.S. exhibited a higher IR of exertional hyponatremia in 2025 compared to their counterparts in other regions, whereas other duty stations including the northeast, midwest, and southern regions showed similar IRs (data not shown). Exertional hyponatremia cases were diagnosed at more than 103 U.S. military installations and geographic locations worldwide during the surveillance period, but 12 U.S. installations contributed 7 or more cases each and accounted for 45.6% of total cases **(Table 2)** . Fort Benning, Georgia, reported 48 cases of exertional hyponatremia, the highest in the Department of War.

**TABLE 2.** Incident Cases of Exertional Hyponatremia by Location of Diagnosis or Report (with at least 7 cases during period of surveillance), Active Component, U.S. Armed Forces, 2021–2025

Location of Diagnosis	No.	% Total
Fort Benning, GA	48	7.88
MCRD Parris Island, SC	27	4.43
NMC San Diego, CA	27	4.43
NMC Portsmouth, VA	22	3.61
Camp Lejeune, NC	17	2.79
Fort Bragg, NC	15	2.46
Walter Reed NMMC, MD	15	2.46
Camp Pendleton, CA	13	2.13
Fort Carson, CO	12	1.97
Fort Hood, TX	11	1.81
JB San Antonio, TX	11	1.81
Fort Shafter, HI	10	1.64
Fort Belvoir, VA	9	1.48
Fort Campbell, KY	9	1.48
JB Lewis-McChord, WA	9	1.48
Camp Foster	8	1.31
Fort Bliss, TX	8	1.31
NAS Jacksonville, FL	7	1.15
Other Locations	331	54.4
Total	609	100.0

Abbreviations: No., number; MCRD, Marine Corps Recruit Depot; JB San Antonio, Joint Base San Antonio; NMC, Naval Medical Center; NMMC, National Military Medical Center; NH, Naval Hospital.

Note: Referral centers include Walter Reed NMMC, NMC San Diego, and NMC Portsmouth.

## Discussion

Since 2023, overall IRs of exertional hyponatremia among U.S. ACSMs have been on a downward trend, with substantial decreases in incidence among Marine Corps members, recruits, those in motor transport occupations, individuals ages 25-29 years, and female service members. Air Force exertional hyponatremia IRs were more variable throughout the 5-year surveillance period.

With the exception of the Air Force, all branches of service evinced downward trends in exertional hyponatremia IRs from 2024 to 2025. Marines and recruits demonstrated particularly sharp declines in exertional hyponatremia cases from 2024 to 2025.

In 2025, incidence of exertional hyponatremia declined for all military occupations, with 2 notable exceptions: health care personnel and pilots and air crew, which had the highest and second-highest IRs, respectively.

Exertional hyponatremia IRs in 2025 decreased among all age groups except for ACSMs ages 30-39 years. The most significant decline was seen in the 25-29-years age group, followed by the 20-24-years age group. A sharp surge in the IR for the ages 35-39-years group reversed the 2024 trend, where the ages 40 years and older group had the highest rate.

In 2022 and 2023, non-Hispanic Black service members had higher IRs, while in 2024 and 2025, non-Hispanic White service members had higher rates. Rates are comparable, however, among racial and ethnic groups. Risk is not definitively linked to any single ethnic group. ^16 - 18^

The IR for hyponatremia in the western U.S. increased in 2025 to become the highest in the nation. Notably, this region contains major military training centers in desert environments, such as the National Training Center at Fort Irwin, California, and the Marine Corps Air Ground Combat Center at Twentynine Palms, California. This report did not assess the intensity of military training or specific climate exposures during the reporting period, however. Consequently, no conclusions can be drawn about the impact of these operational and environmental factors on the observed increase.

Several important limitations should be considered when interpreting the results of this analysis. First, there is no diagnostic code specific for exertional hyponatremia. This lack of specificity may result in inclusion of some non-exertional cases of hyponatremia, thus overestimating the true rate. Consequently, the results of this analysis should be considered estimates of the actual incidence of symptomatic exertional hyponatremia from excessive water consumption among U.S. military members.

In addition, the accuracy of estimated numbers, rates, trends, and correlates of risk depends on the completeness and accuracy of diagnoses that are documented in standardized records of relevant medical encounters. Nonetheless, the decline in the number of diagnoses presenting with exertional hyponatremia may reflect increased awareness, concern, and aggressive management of early cases by military supervisors and primary health care providers. Finally, recruits were identified using an algorithm based on age, rank, location, and time in service, which was only an approximation and likely resulted in some misclassification of recruit training status.

The downward-trending IR for hyponatremia during the surveillance period may be the result of evidence-based policies implemented by the service branches following a period characterized by a surge, particularly among recruits, in the incidence of exertional hyponatremia. The Army's policy, TRADOC Regulation 350-29, was developed to ensure heat illness protocols were more specific and scientifically grounded. ^19^ This policy, reflecting the latest medical knowledge and lessons about exertional illnesses, established a more effective system for managing heat illness and led to more scientific hydration and work protocols, more precise field diagnosis, improved individual risk assessment, and enhanced leader confidence. The Army protocol combines scientific risk management using Wet Bulb Globe Temperature, strict regulations like fluid intake limits, and targeted education to empower leaders and individual soldiers to prevent the condition. Further investigation and monitoring are needed to enhance effective management of exertional hyponatremia within the Air Force, the only service branch without a recent decline in hyponatremia incidence. The Air Force uses its own heat safety manual, DAFMAN 48-151, ^20^ which is based on the same scientific principles as the Army's TRADOC 350-29 ^19^ but is adapted for its specific operational environment.

The high rates of hyponatremia in 2 seemingly low-risk professional categories, health care and pilots and air crew, may stem not from routine operational duties but instead from unique, high-stress training regimens. Notably, motor transport occupations had 0 cases, underscoring that risk is most concentrated in prolonged, individual physical exertion rather than duties centered on vehicle-based operations.

The surge in hyponatremia incidence among ACSMs ages 30-39 years highlights the need for continuous monitoring and investigation into military-specific circumstances. ^21^ Aging is a known risk factor for hyponatremia, and this sudden spike in a typically lower-risk group suggests a possible link to a specific event, such as a unit deployment to a hot environment or a new high-intensity training program for mid-level officers. The fact that the significant decline in the hyponatremia IR among female service members from 2023 to 2025 demonstrated distinct fluctuations suggests an opportunity for in-depth investigation. Published studies present conflicting results on the association between sex and hyponatremia, ^16 , 17 , 21 - 24^ and sustained monitoring among ACSMs could help clarify the influence of biological sex while improving prevention of exertional hyponatremia.

Proper hydration strategies and effective collaborative management, guided by current policy, are crucial for preventing exertional hyponatremia **(Table 3)** . To reduce risk of exertional hyponatremia, service members of all ranks should be cognizant of mitigation measures such as fluid and electrolyte replacement guidelines, identification of high-risk individuals, and the importance of vigilance during associated activities. ^19^ Prevention strategies for exertional hyponatremia, developed for the unique physical demands and environmental exposures of military personnel, should be applied universally to all service members. To resolve scientific ambiguities and protect the health of all service members, collection of detailed data on all cases of exertional hyponatremia is crucial, to inform future analysis and more accurately identify trends and risk factors.

**TABLE 3.** TRADOC Recommendations ^a^ for Continuous Work Duration and Fluid Replacement in Warm and Hot Environments

Easy Work		Moderate Work		Heavy Work		Very Heavy Work	
Heat Category	WBGT Index (°F)	Work (min.)	Water Intake (qt / hr)	Work (min.)	Water Intake (qt / hr)	Work (min.)	Water Intake (qt / hr)	Work (min.)	Water Intake (qt / hr)
1 (White)	78–81.9	NL ^b^	½	NL ^b^	¾	110	¾	45	¾
2 (Green)	82–84.9	NL ^b^	½	NL ^b^	1	70	1	40	1
3 (Yellow)	85–87.9	NL ^b^	¾	NL ^b^	1	60	1	25	1
4 (Red)	88–89.9	NL ^b^	¾	180	1¼	50	1¼	20	1¼
5 (Black)	>90	NL ^b^	1	70	1½	45	1½	20	1½

Notes:
Applies to average-sized and heat-acclimatized service member wearing the operational camouflage pattern uniform. Fluid needs can vary based on individual differences (± ¼ qt/hr) and exposure to full sun or shade (± ¼ qt/hr). CAUTION: Hourly fluid intake should not exceed 1½ qts. CAUTION: Daily fluid intake should not exceed 12 qts.

Abbreviations: TRADOC, Training and Doctrine Command; WBGT, wet bulb global temperature; F, Fahrenheit; min., minimum; qt, quart; hr, hour; NL, no limit.

a Reference 13, page 24.

b No work limit per hour, up to 4 continuous hours.
